# Wet Laboratory Tools Widely Used in Plant Genomics

**DOI:** 10.1155/2009/321975

**Published:** 2010-02-22

**Authors:** Hikmet Budak, Hongbin Zhang, Pushpendra K. Gupta, Boulos Chalhoub, Andrew James, Chunji Liu

**Affiliations:** ^1^Biological Sciences and Bioengineering Program, Faculty of Engineering and Natural Sciences, Sabanci University, 34956 Tuzla, Istanbul, Turkey; ^2^Department of Soil and Crop Sciences, Texas A&M University, College Station, TX, USA; ^3^Ch. Charan Singh University, India; ^4^Institut National de la Recherche Agronomique, France; ^5^Centro de Investigacion Cientifica de Yucatan, Mexico; ^6^Commonwealth Scientific and Research Organization, Australia

The availability of laboratory tools is essential for advanced research in all areas of biological sciences. The recent development of genomic tools has made it possible to deeply investigate and to continuously improve agronomically important traits such as crop yield, quality, and biotic and abiotic stress tolerances. Integrating the newly advanced or developed wet laboratory tools that are widely used in modern genomics research and making them readily accessible will be greatly helpful for research of plant genomics and other disciplines of plant biology. Furthermore, the compilation of the tools will also facilitate scientists to advance the existing tools or develop new tools to address complicated or new questions that were previously intractable in plant genomics and biology. 

In this special issue of the International Journal of Plant Genomics, “*Wet lab tools widely used in plant genomics*”, we present the current status of widely used genomics tools and update them with their new advances. Articles published in this special issue cover tools for structural, functional, and comparative genomics and proteomics. The issue also summarizes the advances of genome technology in the past decades and synthesizes the current status of knowledge of new tools with an extension of suggestions. By covering up-to-date genomics tools, this special issue provides a reference for studying the structural and functional organization and evolution of plant genomes. We aim that this special issue will become useful material for teaching and research in plant genomics and biology. 

There are 8 articles in this special issue, starting with articles on tools for studying plant authopagy (Mitou et al.) and microRNA identification (Unver and Budak), cloning of small RNA (Eric et al.), and the use of virus-induced gene silencing techniques (Unver et al.) for functional analysis of genes and QTLs in plants. Included is also a comprehensive article on heterologous gene expression techniques given by Filiz and Sayers. An up-to-date protocol of Agro-mediated gene transfer in cereal crops is presented by Hensel et al. Additionally, Dechyeva and Schmidt reported one of the critical tools, the molecular cytogenetic mapping of chromosomal fragments and immunostaining of kinetochore proteins, which will greatly help cytogeneticists for identifying and tagging genes in plants, thus promoting plant molecular breeding. 

